# Lingual metastasis as an initial presentation of renal cell carcinoma: a case report

**DOI:** 10.1186/s13256-017-1470-5

**Published:** 2017-11-07

**Authors:** Hanan Raiss, Sophie Duplomb, Sophie Tartas, Mohamed Layachi, Hassan Errihani

**Affiliations:** 1 Service d’Oncologie Médicale, Institut National d’Oncologie, CHU Rabat and Université Mohamed V, Avenue Allal Al Fassi, Madinat Al Irfane, 10000 Rabat, Morocco; 2 Institut de Cancérologie, Hospices Civils de Lyon and Université de Lyon, 165, Chemin du Grand Revoyet, 69495 Pierre-Bénite cedex, France

**Keywords:** Renal cell carcinoma, Tongue metastasis, Lingual metastasis, Sarcomatoid component

## Abstract

**Background:**

Renal cell carcinoma is the third most common tumor that metastasizes to the head and neck, after breast and lung carcinomas. Tongue metastasis as an initial presentation of renal cell carcinoma is extremely rare, and very few cases have been reported. The prognosis is poor. We present a rare case of metastatic renal cell carcinoma that initially presented as a tongue lesion.

**Case presentation:**

We report the case of a 55-year-old white man who presented with a large exophytic lesion on his tongue. A biopsy was taken, and pathologic examination showed a poorly differentiated carcinoma including a sarcomatoid component. Subtotal glossectomy with neck dissection were planned, but a positron emission tomographic-computed tomography scan showed a left kidney mass. Immunohistochemical evaluation of the tongue lesion was performed, and it was compatible with metastasis from primary renal cell carcinoma. The biopsy of the renal lesion showed a high-grade unclassified renal cell carcinoma. Although our patient underwent systemic therapy, he died of hemorrhagic complications 3 months after the initiation of therapy.

**Conclusion:**

Tongue lesions require a complete assessment to distinguish a metastasis from a primary cancer in order to give the appropriate treatment.

## Background

Lingual metastases are extremely rare, accounting for less than 1% of all malignant tongue lesions [1]. Renal cell carcinoma (RCC) is the third most common tumor that metastasizes to the head and neck [2]. The most commonly affected organs are the paranasal sinuses, larynx, jaws, temporal bones, thyroid gland, and parotid glands [3]. Very few cases have presented initially as a tongue metastasis before the diagnosis of primary RCC [4]. Proposed mechanisms for RCC metastasis involve the arterial, venous, or lymphatic circulation. Hematogenous spread appears to be the most common mechanism of distant metastases. RCC invades the local vascular network of the kidney, spreading through the systemic circulation. Head and neck metastases are mostly related to lung metastases. In the absence of pulmonary metastases, spread can be explained through Batson’s venous plexus. This valveless system offers little resistance to the spread of tumor emboli, allowing retrograde flow from the abdominal cavity [1, 5].

The prognosis of metastatic RCC is poor and 5-year survival is less than 10% [5]. Treatment of tongue metastases is usually palliative [6], including surgery, radiotherapy, and systemic therapy. Surgical excision may be proposed to provide short-term palliation of the symptoms and patient comfort [2]; this can be followed by adjuvant radiotherapy to achieve local control of the disease [6]. Systemic therapy for patients with metastatic RCC was previously limited to cytokine therapy with high-dose interleukin-2 (IL-2) and interferon-alpha (IFN-α) [7]. In recent years, several new therapies have become available. These include multitarget tyrosine kinase inhibitors (sunitinib, sorafenib, axitinib, pazopanib, cabozantinib, lenvatinib), monoclonal vascular endothelial growth factor (VEGF)-antibody (bevacizumab), mammalian target of rapamycin (mTOR) inhibitors (everolimus and temsirolimus), and immunotherapy (nivolumab). Thanks to these new therapies, the prognosis of patients with metastatic RCC has improved considerably [8].

We present a case of metastatic RCC that initially presented as a tongue lesion and we discuss the clinical aspects, the difficulties in diagnosis, and the therapeutic options in light of the recent literature.

## Case presentation

**Fig. 1:**
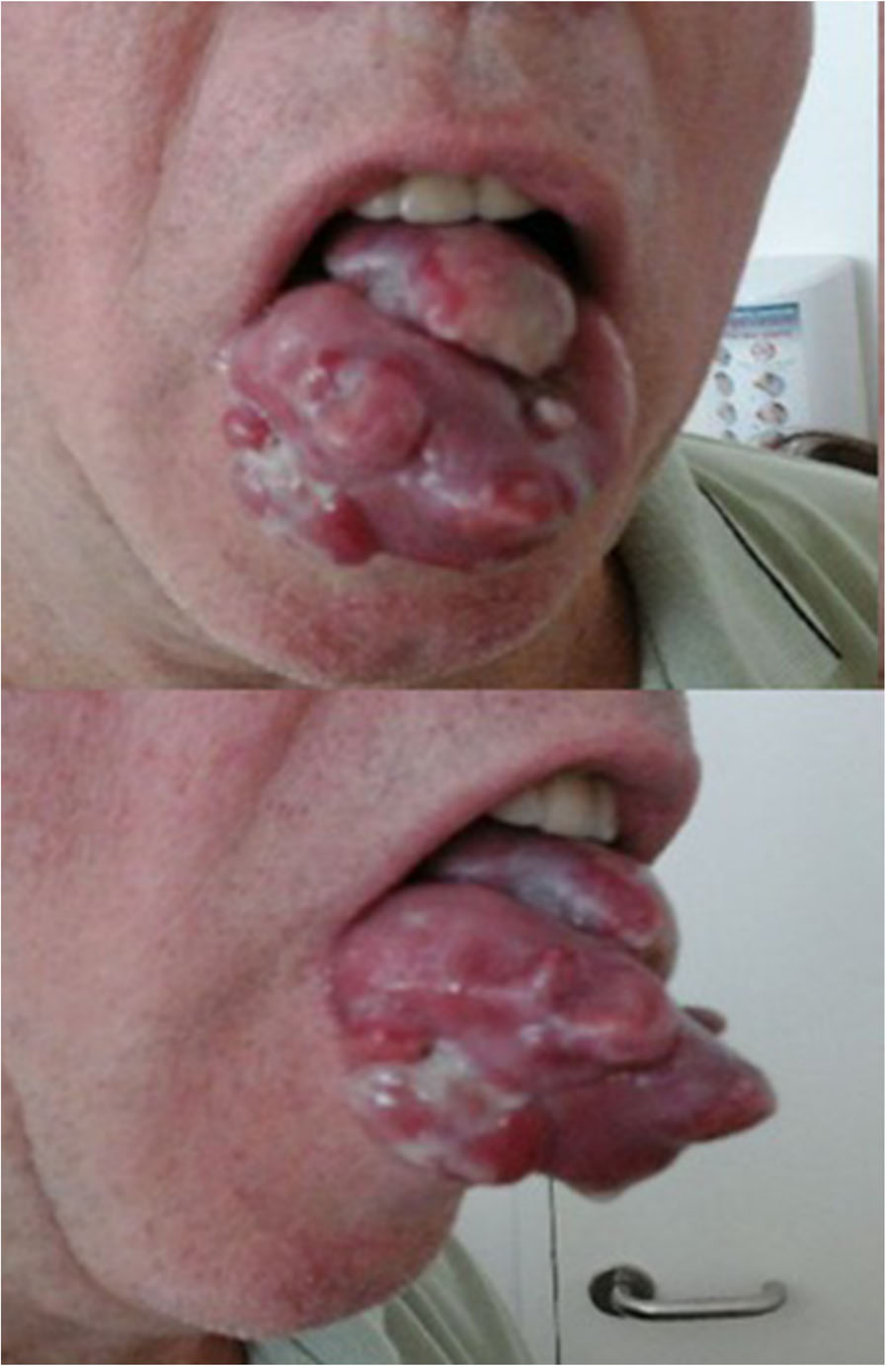
Tongue lesion before treatment

**Fig. 2:**
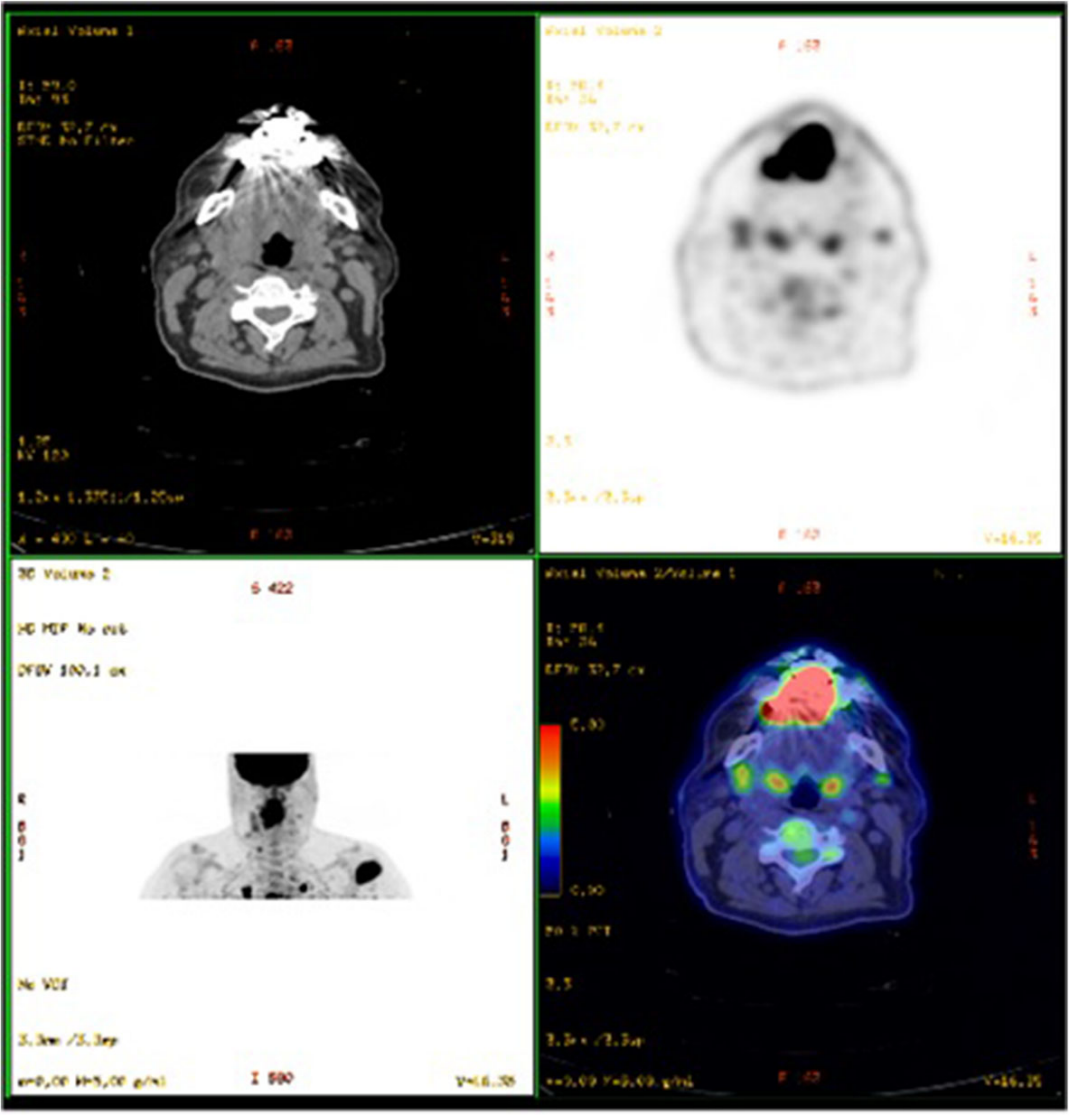
Positron emission tomographic-computed tomography scan shows a tongue lesion

**Fig. 3:**
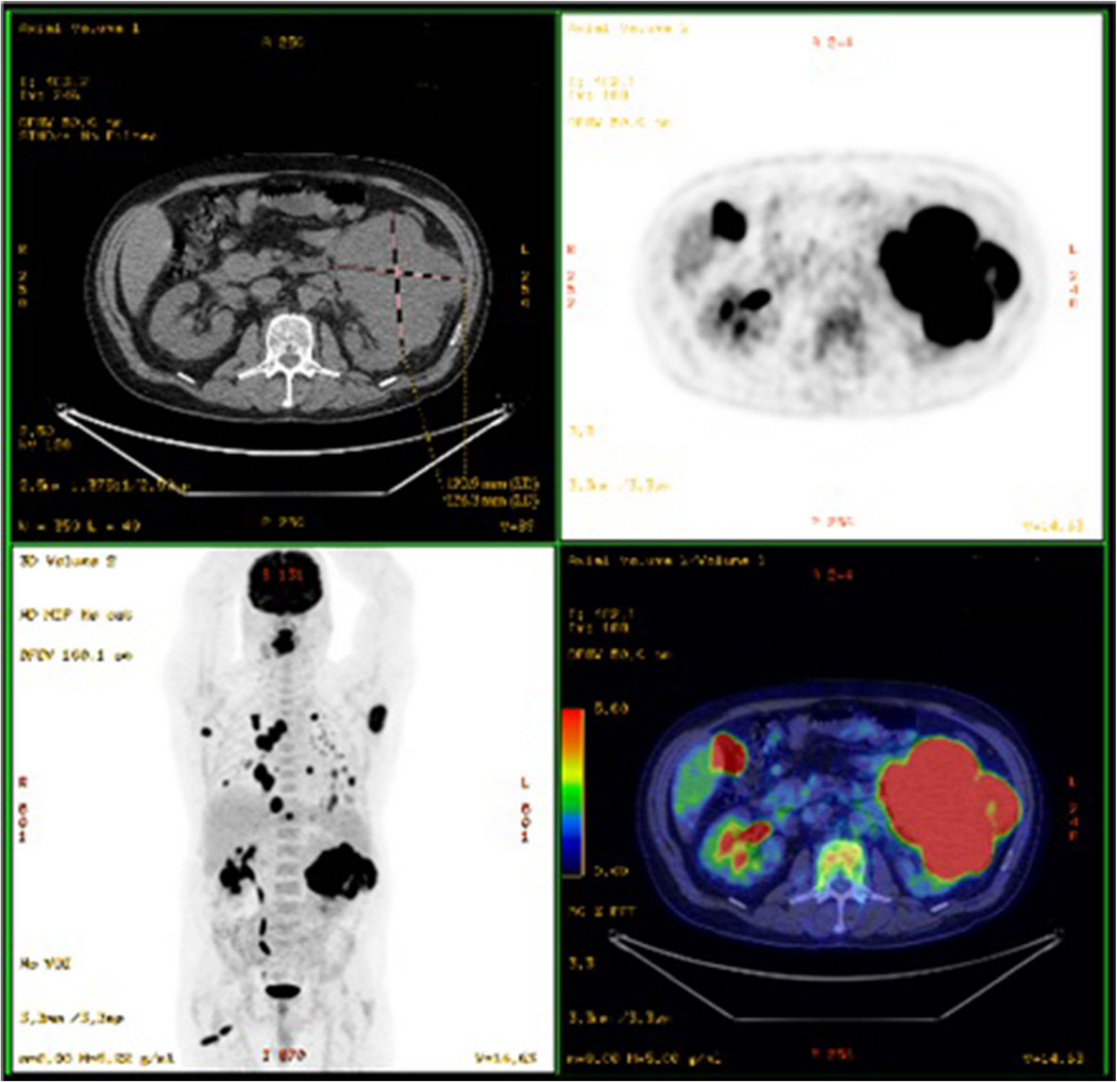
Positron emission tomographic-computed tomography scan shows a left kidney mass

A 55-year-old white man, who was a chronic tobacco smoker, came to our otorhinolaryngology department because of a pedunculated painful lesion on his tongue. He was married and was living with his wife. His medical history included hypertension which is controlled with an angiotensin-converting enzyme inhibitor. On clinical examination, he had a performance status of 1, his blood pressure was 120/70 mmHg, temperature was 37.2 °C, and heart rate was 90 beats/minute with negative dipstick. An oral examination showed a large exophytic lesion occupying the anterior two thirds of the right side of his tongue (Fig. 1). Neurological examination, fiber optic nasopharyngoscopy, and laryngoscopy were normal. Complete blood count was normal with a hemoglobin level of 12 g/dL, a platelet count of 300 × 10^9^/L, and white cell count of 5 × 10^9^/L. His renal function was normal with a creatinine of 0.8 mg/dL. His corrected serum calcium was 2.39 mmol/L and lactate dehydrogenase (LDH) level was 202 UI/L. Aspartate aminotransferase, alanine aminotransferase, and bilirubin were normal. A biopsy of the lesion was taken. Pathologic examination showed a poorly differentiated carcinoma, including a sarcomatoid component represented by spindle cells. Subtotal glossectomy with neck dissection were planned after a staging positron emission tomographic-computed tomography (PET-CT) scan. The PET-CT scan revealed a tongue lesion, bilateral pulmonary metastases, muscle metastases, and a left kidney mass (Figs. 2 and 3). An abdominal computed tomography (CT) scan confirmed the presence of a 13 cm left kidney mass.

Our patient was referred to our oncology department. A detailed medical history was performed, and he described an episode of macroscopic hematuria. A physical examination revealed a subcutaneous nodule in his right axillary region. He had a performance status of 1, and arterial pressure was normal. An immunohistochemical evaluation was performed, and it was positive for Pax 8, CD10, and AE1/AE3 and negative for CK20, CK7, and thyroid transcription factor 1 (TTF1). This result is compatible with a metastasis from a primary renal cell cancer. A biopsy of the renal lesion was performed. Morphological and immunohistochemical findings were consistent with a high-grade unclassified RCC.

**Fig. 4:**
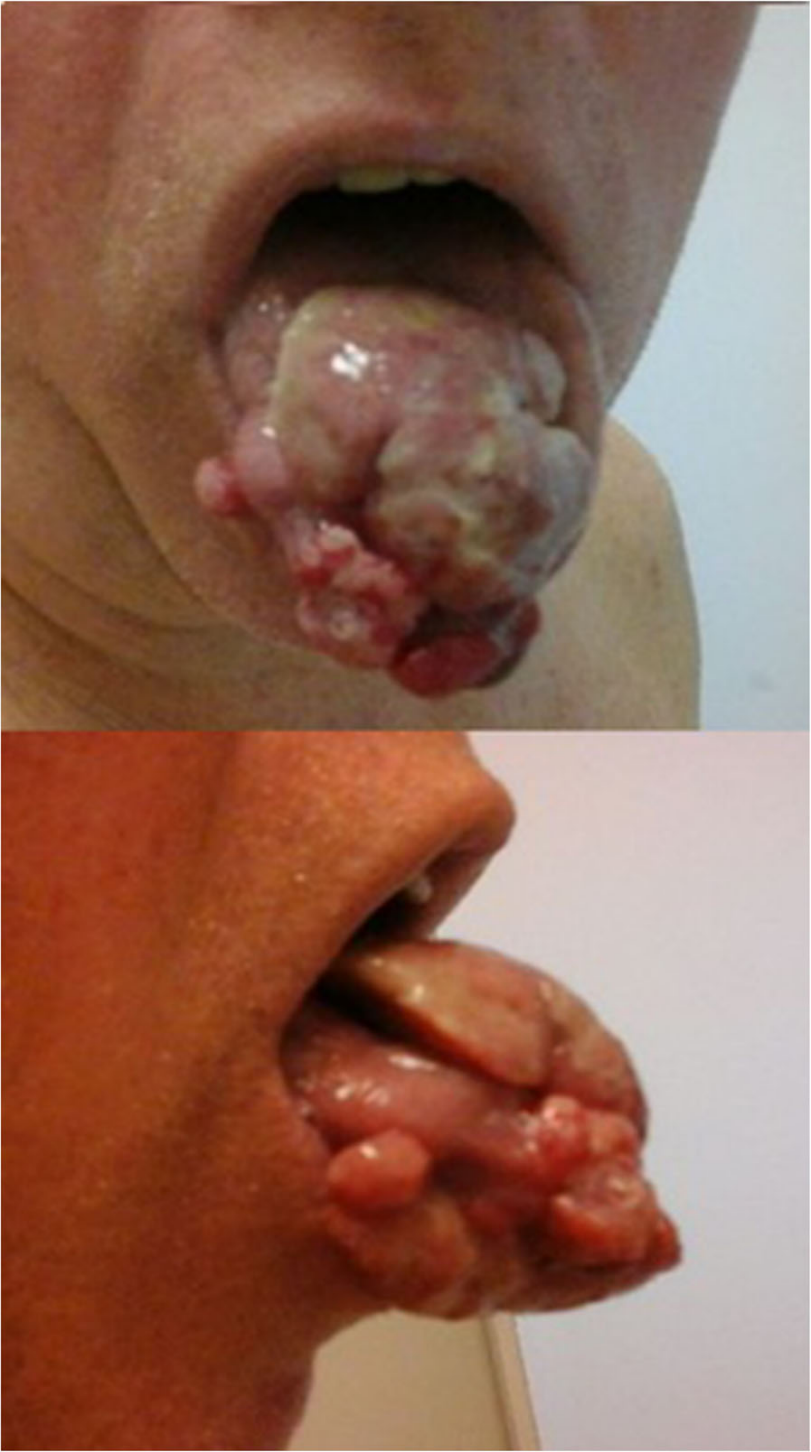
Tongue lesion after 2 weeks of sunitinib treatment

The tongue lesion doubled in size within 2 weeks. Treatment was initiated with sunitinib 50 mg/day, on a schedule of 4 weeks on treatment followed by 2 weeks off. After 2 weeks of treatment, the size of the tongue lesion as well as the size of a subcutaneous nodule had decreased a little (Fig. 4). Sunitinib treatment was discontinued after one cycle because of an unexplained fever. Subsequently, the mass on our patient’s tongue grew and protruded outside his mouth, and caused difficulty in swallowing. CT imaging showed progression of the disease on his tongue, lung, lymph nodes, and muscle. He started second-line systemic therapy with doxorubicin and gemcitabine, but he died 10 days later because of a hemorrhagic complication of the tongue lesion.

## Discussion

RCC is one of the most common tumors to metastasize to the head and neck region, after lung and breast cancer [2, 4, 5]. A tongue metastasis as an initial presentation of RCC is extremely rare [4, 5]. An exhaustive literature review conducted by Azam *et al*. showed 28 reported cases of RCC tongue metastases from 1911 to 2008 [5]. Out of these, three cases presented initially with tongue metastases [3, 5]. To complete this study, we conducted an additional literature review, presented in Table 1, of 18 cases published from 2008 to 2017 [3, 4, 6, 9–23]. Only 6 of the 18 reported cases of RCC tongue metastasis presented initially with tongue metastases [3, 4, 12, 17, 18, 20].

**Table 1:** Previous case reports of lingual metastasis from renal cell carcinoma

Author/year	Age/sex	Presentation	Other metastases	Treatment	Survival (months)
Novák *et al*., 2009 [9]	63/F	History of nephrectomy for RCC 7 years previously. Mass on the tongue	Not known	Not known	Not known
Altinel *et al*., 2010 [4]	67/M	Tongue lesion	None	Excision. Nephrectomy. Interferon-alpha	Not known (alive)
Wadasadawala *et al*., 2011 [10]	48/M	History of nephrectomy and radiotherapy for RCC. Dysphagia and dysphonia and mass on the tongue 5 years later	Adrenals, lungs, mediastinum, vhbone	Radiotherapy	Not known (alive)
Morvan *et al*., 2011 [11]	48/M	History of nephrectomy 3 years previously. Mass on the tongue	Left surrenal, L5 vertebra and left submandibular adenopathy	Excision. Radiation therapy on L5. Sunitinib	60 (alive)
Yoshitomi *et al*., 2011 [12]	47/M	No history of malignant disease. A lump on the tongue	Adrenal gland, pleura, lungs	Nephrectomy. Excision. Interferon. Sunitinib and sorafenib later	24 (alive)
Ghazali *et al*., 2012 [13]	64/F	History of nephrectomy for RCC 14 years previously. Lump on the tongue	Contralateral kidney	Excision	5
Tunio *et al*., 2012 [14]	35/M	History of nephrectomy for RCC. Painless swelling in right lateral tongue	Lung	Excision. Radiotherapy. Intra-oral electron therapy. Sunitinib	Unknown
Novák *et al*., 2012 [15]	63/F	History of nephrectomy for RCC 7 years earlier. Mass on the tongue	None	Cryosurgical therapy	12
Ganini *et al*., 2012 [16]	70/M	History of nephrectomy for RCC. 16 years later, lesion of the hemi-tongue	Adrenals, lymph nodes, lung and bone, skin	Embolization of the left lingual artery	1
Balliram *et al*., 2012 [17]	72/M	Painless lesion of the tongue	Lung	Nephrectomy	3
Ray *et al*., 2013 [3]	65/M	Lesion of the tongue with history of reddish discoloration of urine	Para-aortic lymphadenopathy, psoas muscle and right dome of diaphragm	Excision. Radiotherapy. Sunitinib	Not known
Mazeron *et al*., 2013 [18]	66/M	Tongue lesion	None	Nephrectomy. Sunitinib. Brachytherapy	41
Matias *et al*., 2013 [19]	47/M	Nephrectomy for RCC, metastasis 2 months later.	Lungs, brain, liver, bone, lymph nodes	Temsirolimus followed by sunitinib. Surgical excision and sorafenib	Not known
Abbaszadeh-Bidokhty *et al*., 2014 [6]	80/M	History of nephrectomy for RCC 4 years previously. Swelling on the tongue	None	Surgical excision. Sunitinib followed by sorafenib	6 (alive)
Khobragade *et al*., 2014 [20]	63/M	Swelling over dorsal surface of tongue with difficulty in swallowing	Lungs	Nephrectomy. Excision. Sunitinib	Not known
Altuntaş *et al*., 2015 [21]	70/M	Renal mass	Lungs, lymph nodes, bone	Interferon alpha followed by partial glossectomy and sunitinib after progression	7 (alive)
Wang *et al*., 2016 [22]	71/F	History of nephrectomy 10 years previously. Tumor on the tongue	Hilar lymph node, lungs	Sunitinib	10 (alive)
Lieder *et al*., 2017 [23]	56/unknown	Nephrectomy for RCC. Mass of tongue	Lung, bones, mediastinal nodes, soft tissue of the finger	Excision radiotherapy	3

*F* female, *M* male, *RCC* renal cell carcinoma

The common clinical presentation includes a rapidly growing mass that often causes hemorrhage [23], as in our case. Other symptoms have been reported, such as dysphagia and dysarthria, which may reflect the mass effect [13].

The main histological subtypes of RCC are clear cell, papillary, chromophobe, collecting duct carcinoma, medullary carcinoma, and unclassified [24]. Unclassified RCC represents approximately 4 to 6% of all RCCs and are classified as high-grade lesions, characterized by the presence of a sarcomatoid component without any recognized epithelial elements [25]. The sarcomatoid is a tumor differentiation which may coexist with any histologic subtype of RCC [24], and the presence of sarcomatoid elements is associated with an aggressive disease course [24, 26]. Our patient had a high-grade unclassified RCC with a sarcomatoid component in the biopsy of the tongue lesion.

Treatment alternatives for a tongue metastasis include local excision to relieve symptoms and to provide patient comfort [2]. This palliative surgery is indicated for a rapidly growing metastatic lesion [2]. The introduction of drugs targeting the VEGF pathway or the mTOR pathway has improved the prognosis of RCC [27]. However, an effective systemic therapy for patients with sarcomatoid metastatic RCC has yet to be elucidated [28]. These patients are usually treated with VEGF-targeted therapies, in fewer cases with chemotherapy, or simply referred for supportive care [27]. A retrospective series by Golshayan *et al*. reported a median overall survival (OS) of 11.8 months and a progression-free survival (PFS) of 5.3 months for patients with sarcomatoid RCC treated with sunitinib [29]. In the series of Molina *et al*., the median survival of the 29 patients who were treated with sunitinib was 10 months and the PFS was 4.4 months [30]. In the Eastern Cooperative Oncology Group (ECOG) trial 8802, assessing the combination of doxorubicin with gemcitabine in 39 patients with locally advanced or metastatic RCC with sarcomatoid features, the median OS was 8.8 months [28]. With regard to immunotherapy, in a randomized, open-label phase III study, nivolumab showed a statistically significant improvement in OS compared with everolimus in patients with advanced or metastatic RCC [31]. However, there was not a specific result reported for patients with sarcomatoid component. Geynisman reported one clinical case in which nivolumab led to a rapid response in papillary RCC with sarcomatoid and rhabdoid features [32]. In a phase I study of atezolizumab, an anti-programmed death-ligand 1 antibody, in metastatic RCC with a poor prognosis including sarcomatoid features, the overall response rate (ORR) was 22%, the median OS was 26.2 months, and the median PFS was 4.2 months [33].

The current case was treated with sunitinib, with a good clinical response after one cycle. Unfortunately, the treatment was discontinued because of an unexplained fever, and the disease progressed. A second-line systemic therapy with doxorubicin and gemcitabine was started, but he died due to a hemorrhagic complication.

## Conclusions

It is important for clinicians to be aware that RCC can lead to lingual metastases. Tongue lesions require a complete assessment to distinguish a metastasis from a primary cancer in order to give the appropriate treatment.

## Abbreviations

CT: Computed tomography
mTOR: Mammalian target of rapamycin
OS: Overall survival
PET-CT: Positron-emission tomographic-computed tomography
PFS: Progression-free survival
RCC: Renal cell carcinoma
VEGF: Vascular endothelial growth factor
